# P-1399. Medication Event Reminder Monitors For Tuberculosis Patients: A Systematic Review

**DOI:** 10.1093/ofid/ofaf695.1586

**Published:** 2026-01-11

**Authors:** Purva Shah, Ilham Zaidi, M I rza Adil A Adil, Jaiprakash Gurav, L V Simhachalam Kutikuppala, Nayanika Tummala, Vidhi Sojitra, Tarun Kumar Suvvari

**Affiliations:** Resident, Internal medicine department, Rochester General HospitaL, NY, New York; Advisor, International Society For Chronic Illnesses, Delhi, Delhi, India; Dehradun Institute of Technology, Gorakhpur, Uttar Pradesh, India; Scholar, Armed Forces Medical College, Pune, Maharashtra, India; Dr NTR University of Health Sciences, Vizag, Andhra Pradesh, India; NYMC St Marys St Clares, Parsipanny, NJ; Kathmandu University, Nepal, Mahakali, Nepal; Squad Medicine and Research (SMR), Amadalavalasa, Andhra Pradesh, India

## Abstract

**Background:**

Tuberculosis (TB) treatment involves prolonged regimens that often lead to poor adherence and unfavorable outcomes. Various strategies, including directly observed therapy (DOT), incentives, and digital adherence technologies (DATs), have been employed to address this challenge. This systematic review evaluates the impact of Medication Event Reminder Monitors (MERM) on medication adherence, clinical outcomes, and satisfaction among TB patients and healthcare providers.

**Methods:**

We systematically searched PubMed, ScienceDirect, DOAJ, Embase, and Web of Science from inception to 16 February 2023. Eligible studies included cross-sectional, cohort, case-control, randomized/non-randomized trials, and qualitative studies. Two authors independently screened the studies and assessed quality using the Cochrane risk-of-bias tool for randomized trials and the Newcastle-Ottawa Scale for observational studies.

**Results:**

Eight studies were included: three clinical trials, four prospective studies, and one cross-sectional study, comprising 76,811 TB patients (31.62% female). MERM was found to be a well-accepted and effective tool for improving adherence. Factors influencing adherence included age, gender, occupation, residence, HIV status, and TB diagnosis type. Studies reported higher treatment success and lower loss to follow-up with MERM compared to DOT.

**Conclusion:**

While MERM shows promise in improving adherence and outcomes, acceptance varies due to factors such as side effects and usability. Its potential is significant, especially in high-burden TB countries, but further research is needed to evaluate long-term impact and optimize implementation strategies.

**Disclosures:**

All Authors: No reported disclosures

